# Influence of patient body weight on the probability of return of spontaneous circulation following out-of-hospital cardiac arrest: an exploratory analysis

**DOI:** 10.29045/14784726.2024.9.9.2.11

**Published:** 2024-09-01

**Authors:** Michael W. Hubble, Ginny R. Kaplan, Melisa Martin

**Affiliations:** Wake Technical Community College, North Carolina, USA ORCID iD: https://orcid.org/0000-0002-4683-3767; Methodist University, North Carolina, USA ORCID iD: https://orcid.org/0000-0002-5915-4974; Methodist University, North Carolina, USA ORCID iD: https://orcid.org/0009-0006-3648-7780

**Keywords:** cardiac arrest, cardiopulmonary resuscitation, emergency medical services, obesity, paramedic

## Abstract

**Introduction:**

In addition to key interventions, including bystander CPR and defibrillation, successful resuscitation of out-of-hospital cardiac arrest (OHCA) is also associated with several patient-level factors, including a shockable presenting rhythm, younger age, Caucasian race and female sex. An additional patient-level factor that may influence outcomes is patient weight, yet this attribute has not been extensively studied within the context of OHCA, despite globally increasing obesity rates.

**Objective:**

To assess the relationship between patient weight and return of spontaneous circulation (ROSC) during OHCA.

**Methods:**

This retrospective study included adult patients from a national emergency medical services (EMS) patient record, with witnessed, non-traumatic OHCA prior to EMS arrival from January to December 2020. Logistic regression was used to evaluate the relationship between patient weight and ROSC.

**Results:**

Complete records were available for 9096 patients, of which 64.3% were males and 25.3% were ethnic minorities. The mean age of the participants was 65.01 years (SD = 15.8), with a mean weight of 93.52 kg (SD = 31.5). Altogether, 81.8% of arrests were of presumed cardiac aetiology and 30.3% presented with a shockable rhythm. Bystander CPR and automated external defibrillator (AED) shock were performed in 30.6% and 7.3% of cases, respectively, and 44.0% experienced ROSC. ROSC was less likely with patient weight >100 kg (OR = 0.709, p <0.001), male sex (OR = 0.782, p <0.001), and increasing age and EMS response time (OR = 0.994 per year, p <0.001 and OR = 0.970 per minute, p <0.001, respectively). Patients with shockable rhythms were more likely to achieve ROSC (OR = 1.790, p <0.001), as were patients receiving bystander CPR (OR = 1.170, p <0.001) and defibrillation prior to EMS arrival (OR = 1.658, p <0.001). Although the mean first adrenaline dose (mg/kg) followed a downward trend due to its non-weight-based dosing scheme, the mean total adrenaline dose administered to achieve ROSC demonstrated an upward linear trend of 0.05 mg for every 5 kg of body weight.

**Conclusions:**

Patient weight was negatively associated with ROSC and positively associated with the total adrenaline dose required to attain ROSC.

## Introduction

Sudden cardiac arrest accounts for more than half of all coronary heart disease deaths in the US, with approximately 347,000 emergency medical services (EMS) responses annually for out-of-hospital cardiac arrest (OHCA) (Benjamin et al., 2017). In addition to certain key interventions, such as bystander CPR and early defibrillation (Stiell et al., 2004), successful resuscitation is also associated with several patient-level factors, including a shockable rhythm (Sasson et al., 2010), younger age (Terman et al., 2015), Caucasian race (Shah et al., 2014) and female sex (Kotini-Shah et al., 2021). An additional patient-level factor that may influence outcomes is patient weight, although this attribute has not been extensively studied within the context of pre-hospital resuscitation.

Obesity, defined as body mass index (BMI) >30, is rapidly increasing globally and in the US in particular (Liu et al., 2021; World Health Organization, 2000). Roughly one-third of the global population is overweight or obese, and by 2035 this proportion is projected to reach 51% (World Obesity Federation, 2023). The economic burden of obesity is substantial and is expected to cost the global economy more than four trillion US dollars in 2035 (World Obesity Federation, 2023). With respect to health outcomes, obesity has been implicated as a major risk factor for cardiovascular disease and cardiovascular disease-related deaths (Dwivedi et al., 2020), as well as all-cause excess mortality (Berrington de Gonzalez et al., 2010). Paradoxically, when measured as BMI, some prior investigations have observed an inverse relationship between obesity and certain outcomes among survivors of in-hospital and out-of-hospital cardiac arrest (Bunch et al., 2008; Gupta et al., 2016; Matinrazm et al., 2018; Testori et al., 2011). This so-called ‘obesity paradox’ suggests that obesity may confer a survival benefit, although other studies have failed to demonstrate such an association (Chavda et al., 2022; Lee et al., 2021).

Unfortunately, all these previous studies share the common limitation of being comprised solely of previously resuscitated patients who survived to intensive care unit admission. Thus, it is unknown if the ‘obesity paradox’ observed among the in-hospital post-resuscitation population extends to patients during the initial pre-hospital phase of resuscitation. To explore this knowledge gap, we examined the relationship between patient body weight and return of spontaneous circulation (ROSC) among a national sample of OHCA patients.

## Methods

### Study setting

With Institutional Review Board (IRB) approval from Methodist University, we conducted a retrospective, cross-sectional analysis using data from ESO, Inc. (Austin, TX), one of the nation’s largest providers of EMS electronic health record systems. We used the 2020 ESO Data Collaborative Annual Research data set, which included records from over 2000 EMS agencies in the US that consented to the release of de-identified data for research purposes.

### Sample selection

We identified patients who sustained an OHCA during the calendar year 2020, based upon a recorded paramedic impression of cardiac arrest. These records were filtered to meet our inclusion criteria consisting of adults (≥18 years) who suffered a witnessed, non-traumatic arrest prior to EMS arrival and who received a resuscitation attempt by EMS. In an effort to exclude the competing risk of mortality due to advanced malignancies and other frailties, patients whose weight was <35 kg were excluded, as were patients missing any relevant data elements.

### Outcome measure

The primary outcome measure was the relationship between patient weight (dichotomised as ≤100 kg and >100 kg) and ROSC of any duration during the pre-hospital phase of resuscitation. We elected to dichotomise at 100 kg because the standard adult dose of adrenaline (1 mg) is equivalent to the paediatric weight-based dose of 0.01 mg/kg at this weight and permits comparison with other weight-based studies in the literature.

### Statistical analysis

Abstracted data were analysed using IBM SPSS Statistics version 28 (IBM Corporation; Armonk, NY) with p ≤0.05 indicating statistical significance. Continuous variables are reported as means and standard deviation (SD), while categorical variables are described as percentages. Continuous variables were analysed using Student’s t-test. Categorical data were evaluated using the chi-squared test, continuity correction or Fisher’s exact test, as indicated. Adjusted odds ratios (OR) were derived via logistic regression to control for potentially confounding variables. These variables were selected *a priori* and included: patient age, sex and non-Caucasian race; aetiology of arrest; shockable presenting rhythm; bystander CPR; AED shock prior to EMS arrival; EMS response time; and placement of an advanced airway of any type.

## Results

**Figure 1:**
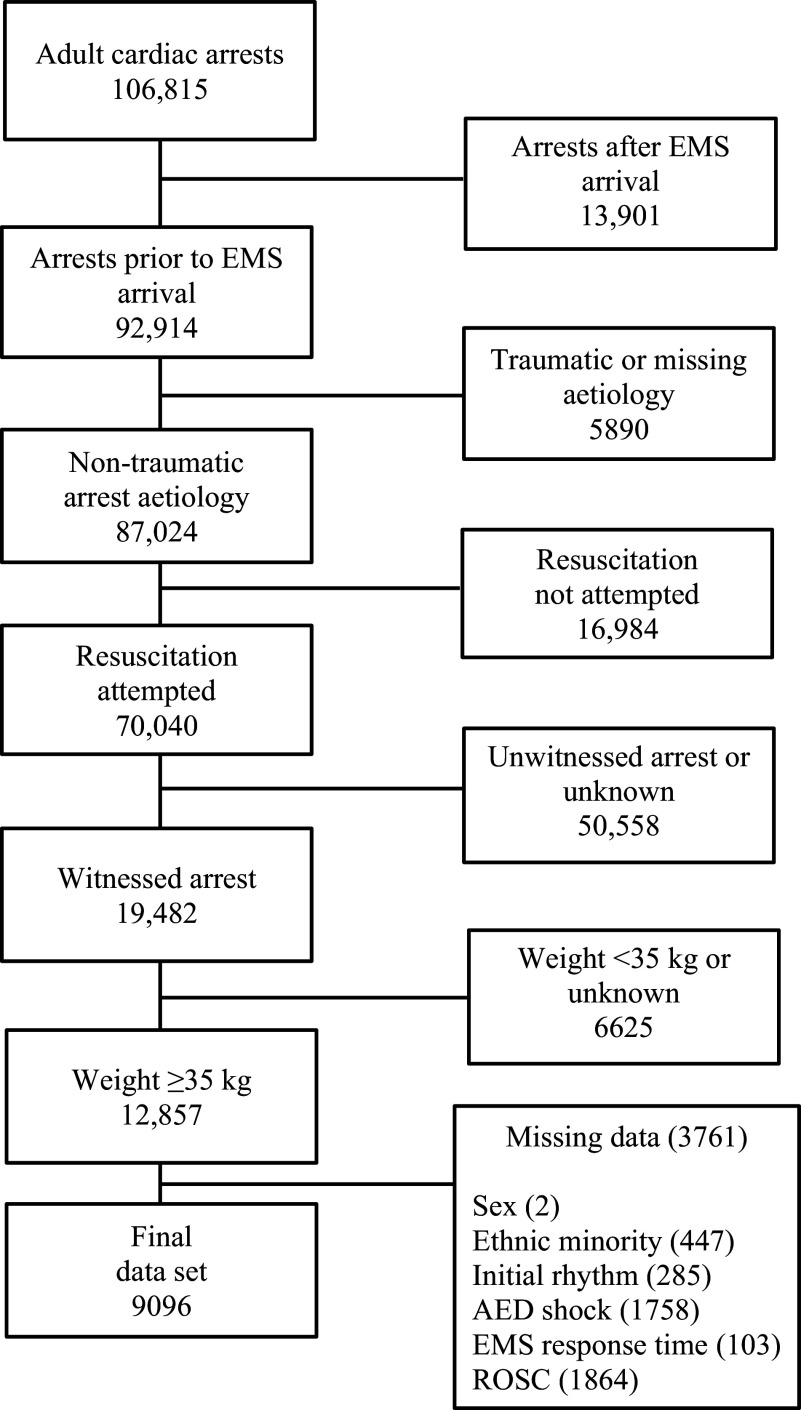
Figure 1. Schematic demonstrating the flow of patients included in the study sample.

During the study period, 106,815 adult patients sustained cardiac arrest, of which 12,857 met inclusion criteria. A total of 3761 were excluded due to incomplete data elements, leaving 9096 patients in the final data set (Figure 1). Of those included in the analysis, males accounted for 64.3% of the sample and 25.3% of the sample were of non-Caucasian race. The mean age of the participants was 65.01 years (SD = 15.8), with a mean weight of 93.52 kg (SD = 31.5). The majority (81.8%, n = 7440) of arrests were of presumed cardiac aetiology, and nearly a third (30.3%, n = 2760) presented with a shockable rhythm. The mean EMS response interval was 8.49 minutes (SD = 5.1), and bystander CPR was performed in 30.6% (2787) of cases. AED defibrillation was attempted prior to EMS arrival in 7.3% (663) of patients, and 78.0% (7099) received an advanced airway at some point during the resuscitation. The mean first and total adrenaline doses were 0.011 mg/kg (SD = 0.004) and 3.81 mg (SD = 2.0), respectively. In total, 3998 (44.0%) experienced ROSC at some point during the pre-hospital phase of resuscitation.

The results of the univariate analysis of patients with and without ROSC are presented in Table 1. Notably, compared to patients without ROSC, a greater proportion of patients with ROSC received bystander CPR (33.1% versus 28.7%, p <0.001) and presented with a shockable rhythm (37.4% versus 24.8%, p <0.001). Patients attaining ROSC also tended to be younger (63.73 versus 66.01 years, p <0.001) and weigh less (90.45 versus 95.93 kg, p <0.001). Patients with ROSC received a lower total adrenaline dose (3.32 versus 4.13 mg, p <0.001) but received a slightly higher weight-adjusted first dose of adrenaline (0.012 versus 0.011 mg/kg, p <0.001). EMS response times were also shorter for those who attained ROSC compared to those who did not (7.97 versus 8.71 minutes, p <0.001). 

**Table 1.** Univariate comparison of those with and without ROSC.

	All patients (N = 9096)	Without ROSC (N = 5098)	With ROSC (N = 3998)	p-value
Age (years)	65.01 (SD = 15.8)	66.01	63.73	<0.001
Male sex	64.3%	66.3%	61.7%	<0.001
Non-Caucasian race	25.3%	25.6%	25.0%	0.477
Mean weight (kg)	93.52 (SD = 31.5)	95.93	90.45	<0.001
Aetiology of arrest: Presumed cardiac Respiratory Drug overdose Other	81.8% 12.1% 2.6% 3.4%	61.4% 51.8% 44.9% 59.2%	38.6% 48.2% 55.1% 40.8%	<0.001
Received bystander CPR	30.6%	28.7%	33.1%	<0.001
Initial shockable rhythm	30.3%	24.8%	37.4%	<0.001
Received AED shock prior to EMS arrival	7.3%	5.3%	9.8%	<0.001
EMS response time (minutes)	8.49 (SD = 5.1)	8.71	7.97	<0.001
Received advanced airway placement	78.0%	78.8%	77.0%	0.042
First adrenaline dose (mg/kg)	0.011 (SD = 0.004)	0.011	0.012	<0.001
Total adrenaline dose (mg)	3.81 (SD = 2.0)	4.13	3.32	<0.001

The results of the univariate analysis of patients by weight class are presented in Table 2. Despite being younger and having a higher proportion of patients with a shockable presenting rhythm, patients weighing >100 kg were less likely to attain ROSC (28.5% versus 46.7%, p <0.001). Heavier patients also had a lower rate of successful first defibrillations, received a higher number of total defibrillations and received a higher cumulative dose of adrenaline. There were no statistically significant differences in the time to place an advanced airway, defibrillate or establish vascular access in the form of an IO or IV placement. There was a statistically significant but likely clinically unimportant difference between the weight classes in the scene arrival to first adrenaline administration interval (9.0 versus 9.3 minutes, respectively).

**Table 2.** Univariate comparison of patients by weight class.

	≤100 kg (N = 6059)	>100 kg (N = 3037)	p-value
Age (years)	66.53	61.98	<0.001
Male sex	59.5%	73.8%	<0.001
Non-Caucasian race	25.9%	24.2%	0.073
Mean weight (kg)	75.93	128.61	<0.001
Aetiology of arrest: Presumed cardiac Respiratory Drug overdose Other	80.8% 12.4% 3.1% 3.7%	83.7% 11.7% 1.7% 2.8%	<0.001
EMS response time (minutes)	8.31	8.54	0.038
Received bystander CPR	30.2%	31.5%	0.229
Used CPR feedback device	37.3%	38.2%	0.443
Initial shockable rhythm	29.3%	32.4%	0.002
Received AED shock prior to EMS arrival	7.1%	7.6%	0.386
Scene arrival to shock for initially shockable rhythms (minutes)	6.17	6.04	0.322
First shock energy (joules)^a^	229.17	230.92	0.595
Proportion of successful first shocks	20.4%	14.6%	<0.001
Total number of shocks per patient	1.07	1.20	0.006
Received advanced airway placement	77.1%	80.0%	0.002
Scene arrival to first advanced airway placement (minutes)	10.22	10.46	0.211
Scene arrival to IV placement (minutes)	11.04	11.79	0.052
Scene arrival to IO placement (minutes)	7.52	7.40	0.822
Scene arrival to first adrenaline dose (minutes)	9.00	9.31	0.047
First adrenaline dose (mg/kg)	0.013	0.004	<0.001
Total adrenaline dose (mg)	3.71	3.99	<0.001
Total adrenaline dose (mg/kg)	0.050	0.318	<0.001
Total on-scene time (minutes)	30.61	32.52	<0.001
ROSC	46.7%	28.5%	<0.001

^a^Data on the specific defibrillator models and defibrillation waveforms used were not available.

**Figure 2:**
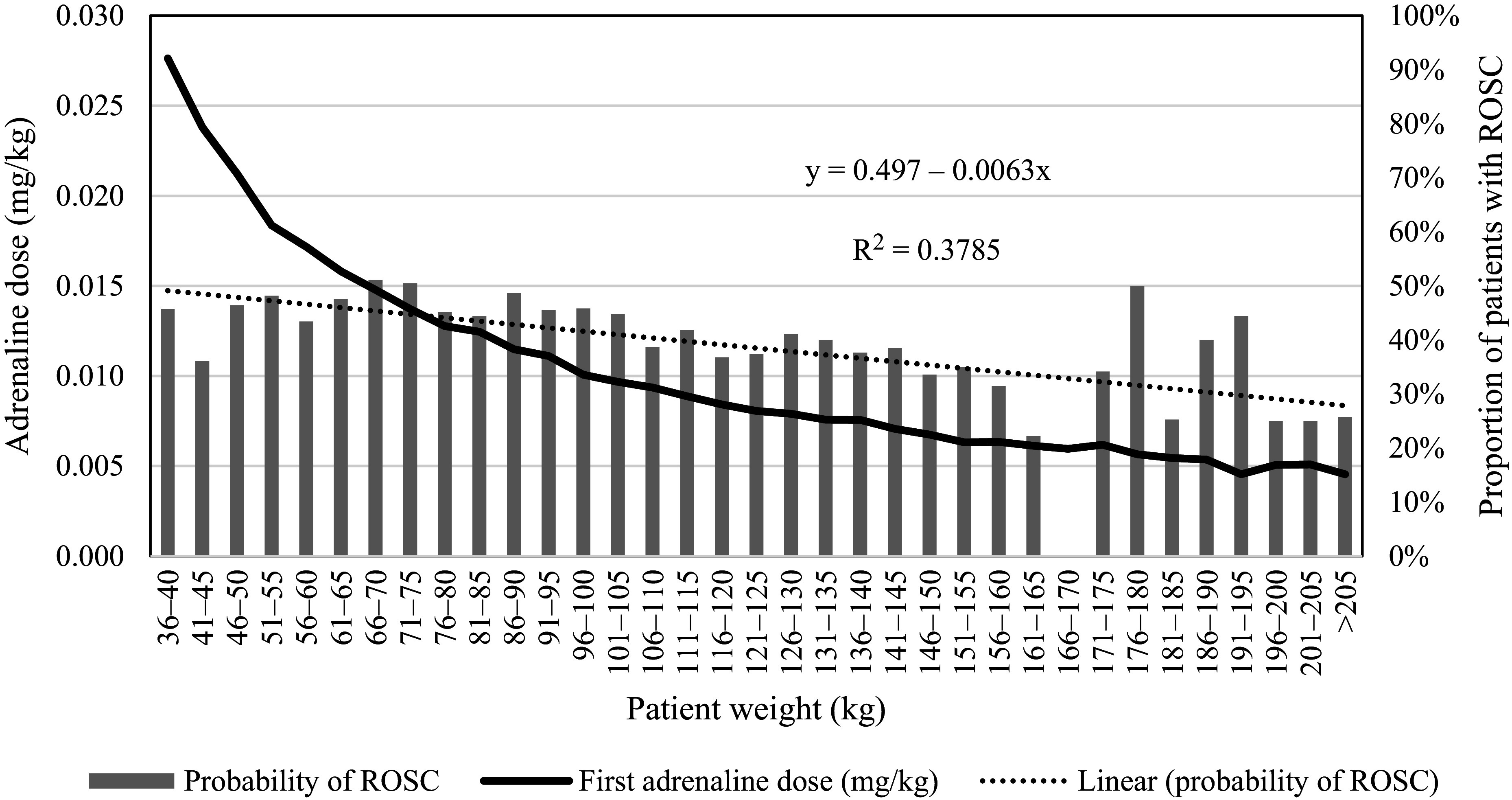
Figure 2. First adrenaline dose (mg/kg) and proportion of patients with ROSC by patient weight class.

A simple linear regression model was used to fit a trend line to the proportion of patients with ROSC for each 5 kg increment of patient weight. Because the regression equation is not weighted by sample size, and is therefore subject to the risk of bias from small sample sizes, all weight categories with fewer than five patients were excluded from this analysis. The regression model indicated that approximately 49% of patients would experience ROSC and that this proportion would decline by approximately 0.6% per 5 kg of body weight (R^2^ = 0.38) (Figure 2).

**Figure 3:**
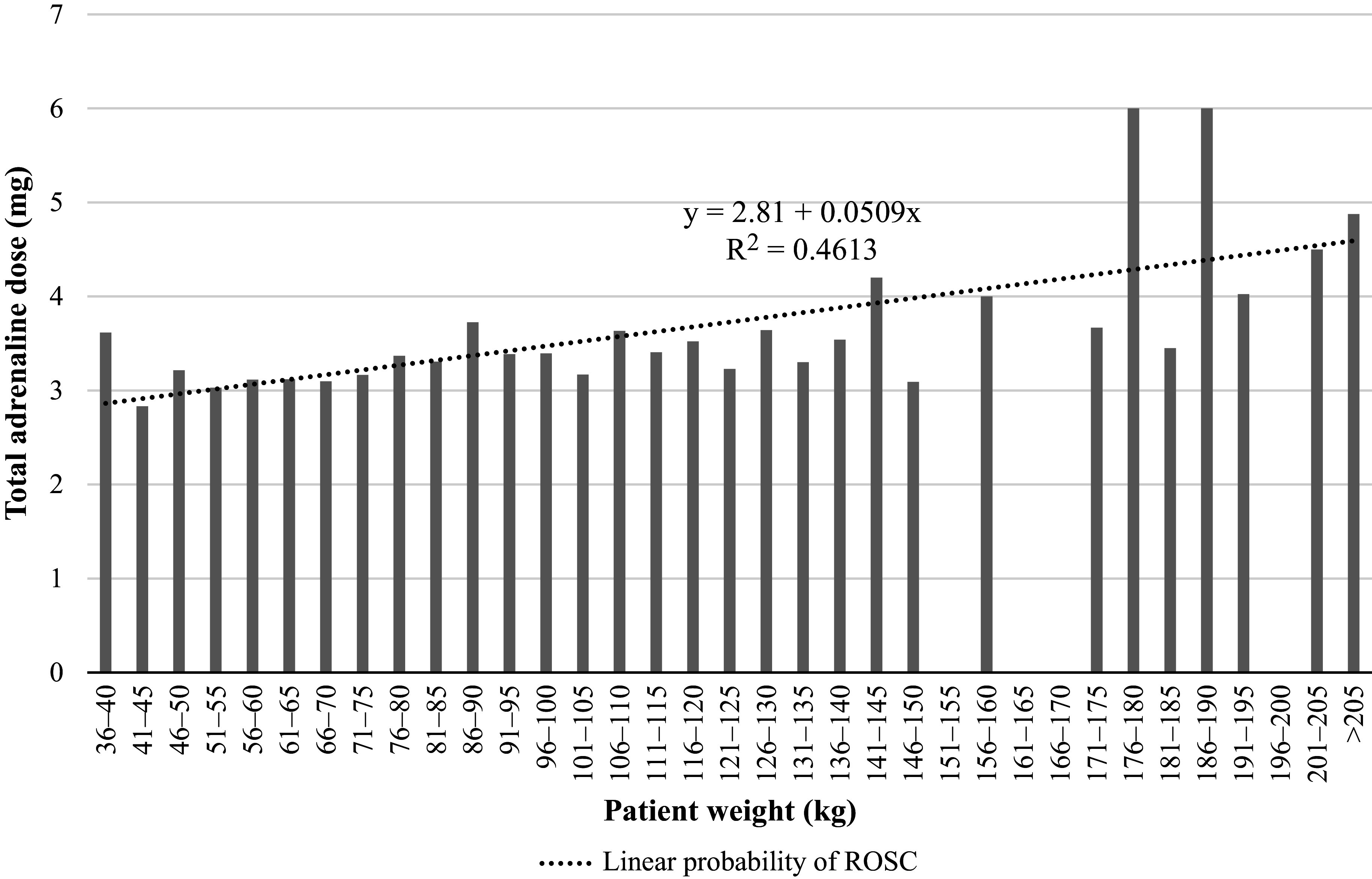
Figure 3. Mean total adrenaline dose administered among patients with ROSC by patient weight class.

Because the mean first adrenaline dose followed a negative trend due to its non-weight-based dosing recommendation (Figure 2), it is unsurprising that the mean total number of doses administered to achieve ROSC increased and followed a linear upward trend of 0.05 mg per 5 kg of body weight (R^2^ = 0.46) (Figure 3).

Logistic regression was used to evaluate the influence of patient weight on ROSC while controlling for potentially confounding variables. ROSC was less likely when patient weight exceeded 100 kg (OR = 0.709, p <0.001), in males (OR = 0.782, p <0.001) and with both increasing EMS response time and age (OR = 0.970 per minute, p <0.001 and OR = 0.994 per year, p <0.001, respectively). Compared to patients with non-shockable rhythms, patients presenting with shockable rhythms were more likely to achieve ROSC (OR = 1.790, p <0.001), as were patients receiving bystander CPR (OR = 1.170, p <0.001) and those who were defibrillated with an AED prior to EMS arrival (OR = 1.658, p <0.001). Advanced airway placement and non-Caucasian race were not independent predictors of pre-hospital ROSC (Table 3).

**Table 3.** Adjusted odds ratio for ROSC.

	Odds ratio	95% CI	p-value
Age (per year)	0.994	0.991–0.997	<0.001
Male sex	0.782	0.714–0.856	<0.001
Non-Caucasian race	0.976	0.883–1.078	0.628
Weight (>100 kg)	0.709	0.646–0.778	<0.001
Aetiology of arrest Presumed cardiac Respiratory Drug overdose Other	(reference) 1.686 2.816 1.074	1.478–1.922 2.103–3.769 0.849–1.358	<0.001 <0.001 0.554
Received bystander CPR	1.170	1.066–1.285	<0.001
Initial shockable rhythm	1.790	1.620–1.977	<0.001
Received AED shock prior to EMS arrival	1.658	1.396–1.969	<0.001
Received advanced airway placement	0.969	0.874–1.075	0.555
EMS response time (per minute)	0.970	0.962–0.979	<0.001

## Discussion

Although some studies have observed a favourable relationship between elevated BMI and survival among post-resuscitation cardiac arrest patients admitted to the ICU, such a relationship has not been adequately investigated within the context of the pre-hospital phase of resuscitation. We sought to address this gap in the literature and found no evidence of the ‘obesity paradox’ among OHCA patients during the pre-hospital resuscitation phase when using weight >100 kg as a surrogate measure of obesity. In contrast, we found that increasing patient weight was independently associated with a reduced likelihood of ROSC after adjusting for a number of potential confounders. Indeed, there are many plausible reasons to suspect that increased patient weight could, in fact, impede the effectiveness of pre-hospital resuscitative efforts. A larger body habitus may pose difficulties in key resuscitative procedures including vascular access, adequate ventilations, placing an advanced airway, positioning defibrillator pads and performing chest compressions. Body habitus may also influence the effectiveness of vasopressors and defibrillation because neither of these interventions are adjusted for differing body weights.

Successful defibrillation is a function of delivered trans-cardiac current. In obese patients, such current delivery may be inhibited by a higher transthoracic impedance (Zelinka et al., 2007). In an attempt to improve defibrillation success, some observers advocate for using higher defibrillation energy to overcome elevated impedance in obese patients (Tacker et al., 1974; Zelinka et al., 2007). In contrast, other studies have demonstrated that biphasic defibrillators can adequately compensate for transthoracic impedance (Ogunnaike et al., 2016; White et al., 2004). In one small study of OHCA patients with an initially shockable presenting rhythm, White et al. (2004) found no difference in mean body weight between those with and without first shock success or pre-hospital ROSC. Additionally, there was only a modest correlation between patient weight and transthoracic impedance. In contrast to White et al. (2004), our dataset revealed a significantly lower proportion of successful first shocks among patients weighing >100 kg (14.6% versus 20.4%, p <0.001), as well as a higher mean cumulative number of shocks per patient (1.20 versus 1.07, p = 0.006). However, we found no difference in the scene arrival to first shock interval among patients with initially shockable rhythms, implying that elevated patient weight does not delay pad placement and initial shock delivery. Due to inherent limitations in our retrospective dataset, we are unable to ascribe causes of the reduced first shock effectiveness, although increased impedance due to body weight and/or poor pad positioning due to body mass are suspect.

Airway and ventilatory management of obese patients can be particularly challenging due to limited neck extension, distortion of oropharyngeal anatomy, mask seal challenges and impaired respiratory physiology. Few studies have investigated patient weight as a risk factor for failed or difficult airway management in the pre-hospital setting. Holmberg et al. (2011) reported an increased difficulty with pre-hospital intubation among patients with extreme obesity compared to lesser degrees of obesity, but there was no such relationship regarding overall intubation success. In contrast, Wang et al. (2003) reported that increased patient weight was associated with an increased frequency of failed intubation, and Gaither et al. (2014) reported obesity to be a contributing factor in 13% of cases of failed pre-hospital intubation. We were unable to identify any studies evaluating the suitability and effectiveness of supraglottic airways among obese patients specific to the pre-hospital setting, although several reports of their use in the operating room suggest that failure rates and complications are low (Natalini et al., 2003; Prabha et al., 2018; Shariffuddin et al., 2020; Weber et al., 2011).

Regarding respiratory physiology, functional residual capacity, expiratory reserve volume, vital capacity, total lung capacity and residual capacity have been shown to decline by 0.5%–5% for each unit increase in BMI (Parker et al., 2019; Salome et al., 2010). Of these, the impact of reduced functional residual capacity is particularly insidious, as it leads to increased airway resistance, atelectasis and intrapulmonary shunting. Obesity is also associated with ventilation–perfusion mismatch and reduced chest wall compliance resulting from an upper lobe ventilatory pattern (Dixon & Peters, 2018). These detrimental effects are worsened when the patient is in a supine position, which is virtually ubiquitous in the OHCA setting.

Unlike previous pre-hospital investigations, we found a statistically significant greater proportion of successful advanced airway placement among the >100-kg group, although the effect size was small. For our purposes, advanced airways included supraglottic airways, surgical airways and endotracheal intubation of any form (direct laryngoscopy, video laryngoscopy, nasotracheal intubation, rapid sequence induction and drug-facilitated intubation). We did not observe any difference in the time required to place an advanced airway. Unfortunately, we lacked the necessary data to evaluate the adequacy of ventilations among our sample.

Rapid vascular access followed by adrenaline administration has been associated with improved OHCA clinical outcomes (Ran et al., 2020). Unfortunately, obesity has been implicated as a maleffect regarding difficulties in obtaining intravenous and intraosseous access (Kehrl et al., 2016; Sebbane et al., 2013). Additionally, the optimum dose of adrenaline for obese patients remains unclear. Dosing recommendations for most medications are developed for non-obese patients and then extrapolated to the obese population, which may potentially result in drug toxicity or treatment failure (Parker et al., 2019). The recommendation for adrenaline originated from a study in the 1960s where 1 mg given to asphyxiated dogs improved survival, but the dose was never weight-adjusted when extrapolated to humans (Pearson & Redding, 1963). Current paediatric advanced life support (PALS) guidelines recommend a weight-based dose of 0.01 mg/kg for paediatric patients weighing less than 100 kg, after which they receive a standard dose of 1 mg, which is equivalent to advanced cardiac life support (ACLS) adult dosing guidelines (Panchal et al., 2020; Topjian et al., 2020). These guidelines ultimately lead to incongruous dosing, whereby a 50-kg adult receives twice the dose of adrenaline as that given to an adolescent of the same weight, yet the same dose as a 200-kg adult. The clinical effects of these dosing differences are also not well described in the literature. In a retrospective analysis of patients receiving the standard 1-mg dose, Wang et al. (2016) reported worse outcomes for patients ≥82.5 kg, suggesting that these patients may not be receiving an adequate dose, yet prior studies failed to identify any benefit of high-dose adrenaline among adults. However, the high-dose adrenaline dosages were much greater (0.2 mg/kg (Brown et al., 1992) and 7 mg (Stiell et al., 1992)) than if applying the standard paediatric weight-based dose of 0.01 mg/kg without the 1 mg maximum dose constraint (e.g. 2 mg for a 200-kg patient) and may have exceeded any marginal clinical benefit.

We did not observe any differences in the time required to establish IV or IO access between weight classes, although there was a very small, and likely clinically insignificant, increase in the average time to deliver the first dose of adrenaline in the >100-kg group. As expected, the average first dose (mg/kg) of adrenaline declined across the weight classes given the standardised 1 mg dosing regimen for adults. However, we did observe that the total dose administered among those attaining ROSC increased linearly with increasing weight. This suggests that the non-weight-adjusted dosing regimen may result in underdosing of larger patients, which is consistent with the conclusions of Wang et al. (2016).

For reasons unknown, our sample experienced a substantially lower rate of bystander CPR (30.6%) compared to national rates (40.2%) reported for the US (Tsao et al., 2023). In addition, whether provided by professional rescuers or bystanders, chest compressions may be less effective among larger patients. Current CPR guidelines recommend placement of the hands on the lower half of the sternum with a compression depth of 5–6 cm (Panchal et al., 2020). However, due to increased adipose tissue of the chest wall and a more cephalad position of the diaphragm, this hand position may not be appropriate for obese patients, and a more cephalad position may be optimal (Lee et al., 2018). Moreover, the 5–6 cm compression depth recommendation is unadjusted for body habitus and is likely insufficient for obese patients (Lee et al., 2015, 2019).

### Limitations

Limitations in our study design and data source warrant caution when interpreting our findings. This study is subject to the usual limitations of retrospective design, including the completeness and accuracy of data reporting. In total, 29% of records were excluded due to one or more missing values. Regarding data accuracy, it is unknown to what extent patient weights were estimated rather than obtained from family members or hospital medical records. Although paramedic estimates were reported to correlate well with hospital measurements among OHCA patients in one small study (Martin et al., 1994), the accuracy of weight estimates in our dataset is unknown. Moreover, without patient height in the dataset, we were unable to compute a BMI, which is a more customary measure of obesity. Thus, we were compelled to use weight >100 kg as a surrogate measure of obesity.

We did not detect any weight-based differences in the time required to attempt defibrillation, airway placement or vascular access. However, we did not have data to evaluate quality measures of on-scene CPR and, presumably, the larger body habitus of some patients may have resulted in lower-quality chest compressions. Consequently, we cannot rule out that variations in CPR quality may have influenced the likelihood of ROSC.

We lacked data on co-morbidities, which tend to be disproportionately higher among obese patients. Thus, how these co-morbidities may have affected our results remains unknown. Additionally, the data were collected during the COVID-19 pandemic, which is associated with worse OHCA outcomes, and this may have influenced our results (Bielski et al., 2021).

Finally, hospital discharge data as well as the timing and duration of ROSC were largely unavailable for this study, and the influence of OHCA patient weight on longer-term outcomes remains unknown.

## Conclusion

Within the limits of our retrospective study design, we found no evidence of the ‘obesity paradox’ during the pre-hospital phase of resuscitation that has been reported among post-resuscitation cardiac arrest patients admitted to the ICU. Instead, we observed that patient weight was negatively associated with ROSC. Patients weighing >100 kg also had a lower success rate of first shocks and received a greater number of shocks in total. In addition, among patients attaining ROSC, those weighing over 100 kg required larger cumulative doses of adrenaline. As such, EMS clinicians should be cognisant of the prognostic implications of body weight and anticipate some of the unique resuscitation challenges posed by this patient population during OHCA.

Our exploratory analysis contributes to the understanding of resuscitating patients with larger body habitus. Additional study is needed to further elucidate the relationship between patient weight and medication dosing, chest compression hand placement, depth, and effectiveness, ventilatory metrics and co-morbidities, as these factors probably play a critical role in attaining ROSC.

## Acknowledgements

The data were provided by ESO, Inc. and the authors wish to express their appreciation to ESO for their assistance. The content derived from this data set remains the property of ESO, Inc. ESO is not responsible for any claims arising from works based on the original data, text, tables or figures.

## Author contributions

Each author contributed to the design of the study. MH conducted the statistical analysis and drafted the manuscript, and all authors contributed substantially to its revision. MH acts as the guarantor for this article.

## Conflict of interest

None declared.

## Ethics

Ethical approval was received from the Methodist University Internal Review Board.

## Funding

None.
